# Effects of early treatment with nonsteroidal anti-inflammatory drugs (NSAIDs) on the bronchoalveolar lavage proteome and oxylipids during bovine respiratory syncytial virus (BRSV) infection

**DOI:** 10.1371/journal.pone.0309609

**Published:** 2024-11-15

**Authors:** Sara Hägglund, Eve Laloy, Ignacio Alvarez, Yongzhi Guo, Gabriella Hallbrink Ågren, Haleh Yazdan Panah, Anna Widgren, Jonas Bergquist, Anna Hillström, Vincent Baillif, Laure Saias, Marc Dubourdeau, Edouard Timsit, Jean François Valarcher

**Affiliations:** 1 HPIG, Ruminant Medicine Unit, Department of Clinical Sciences, Swedish University of Agricultural Sciences, Uppsala, Sweden; 2 Laboratoire VETODIAG, Saint-Pierre-en-Auge, France; 3 Department of Chemistry-BMC, Analytical Chemistry and Neurochemistry, Uppsala University, Uppsala, Sweden; 4 Department of Animal Biosciences, Swedish University of Agricultural Sciences, Uppsala, Sweden; 5 Clinical Pathology Laboratory, University Animal Hospital, Swedish University of Agricultural Sciences, Uppsala, Sweden; 6 Ambiotis SAS, Toulouse, France; 7 CEVA Santé Animale, Libourne, France; Michigan State University, UNITED STATES OF AMERICA

## Abstract

Non-steroidal anti-inflammatory drugs (NSAID) are not recommended for use against pneumonia in humans, but are commonly utilised against bovine respiratory disease. This study aimed to determine if the use of NSAIDs in the early phase of bovine respiratory syncytial virus (BRSV)-infection limits pulmonary inflammation. Four to nine-week old calves were infected with BRSV by aerosol and were treated with either meloxicam intravenously on day (D)4 (n = 5, MEL), acetylsalicylat-DL-lysin intravenously on D4 and D5 (n = 5, ASA), or were left untreated as controls (n = 5, CTR). Clinical signs were monitored daily until necropsy on D7, BRSV-RNA was detected in nasal swabs and bronchoalveolar lavage (BAL) by RT-qPCR, inflammatory cells and proteins were identified in BAL by cytology and label-free quantitative mass spectrometry-based proteomics, respectively, and oxylipids were quantified in BAL and plasma by liquid chromatography tandem mass spectrometry with triple quadrupole mass detectors. The calves developed mild to moderate signs of respiratory disease and, with the exception of one MEL-treated and one ASA-treated calf, limited lung lesions. None of the treatments had a significant effect on virus replication, clinical signs or lung lesion extent. Relative to controls, both treatments initially induced a downregulation of proteins in BAL. Immunoglobulin (Ig)-related proteins, such as the Ig kappa and lambda locus and the joining chain of IgA and IgM, were downregulated in MEL-treated calves compared to controls. In addition, meloxicam induced an increased neutrophil influx in BAL in response to BRSV, possibly related to a reduction in plasma prostaglandin, and to a downregulation of The Liver X Receptor/ Retinoid X Receptor (LXR/RXR), the Farnesoid X Receptor (FXR)/RXR and the 24-Dehydrocholesterol Reductase (DHC24) signalling pathways in the lung. The risk of NSAIDs to increase neutrophil activity during stimulation with BRSV or other toll-like receptor 4 agonists needs to be investigated further. Since augmented neutrophil responses can be detrimental, the results of the present study do not support the use of NSAIDs to prevent the clinical expression of BRSV-infection.

## Introduction

Respiratory disease is a major problem and a common reason for antimicrobial treatment in intensively reared calves. Besides immediately affecting animal welfare, the presence of lung lesions can have long term consequences, such as impaired growth and an increased risk of culling before first parturition [1–3]. The aetiology of the disease is multifactorial: it involves pathogens as well as factors related with environment and husbandry, including stress and insufficient passive immunity. Viral infections often precede bacterial infection of the lung, but bacteria seem also to act alone, because the usage of antibiotics in a very early stage leads to an improved recovery [4, 5]. However, a large proportion of animals with early clinical signs are also able to heal spontaneously and the application of a too prompt treatment is therefore not the most sustainable strategy. Antibiotics needs to be administered in time to avoid extensive lesions and excessive inflammation but should be reserved to individuals that do not recover quickly on their own [6].

Along with the application of stricter policies regarding antibiotic use in Europe, non-steroidal anti-inflammatory drugs (NSAIDs) appear more often used alone and in the early stages of respiratory disease in cattle. These drugs decrease the rectal temperature, but very few studies show any effect on the neutrophil influx in the lung and lung lesions [7]. Some studies demonstrated that treating calves with NSAIDs alone at the very early stage of the disease can increase the risk for repeated periods of disease, compared to treating with antibiotics [5, 8]. However, it is difficult to conclude whether NSAIDs alone is better or worse than no medical treatment at all, because untreated control animals were not included in these studies.

In contrast to the extensive use of NSAIDs in cattle, NSAIDs are not included in the USA or European guidelines on the management of community-acquired pneumonia in humans [9]. This is mainly due to digestive and cardio-vascular adverse effects, combined with a lack of effect against respiratory symptoms [10]. Furthermore, worsened complications from pneumonia have been reported with NSAID treatment [11, 12], likely because i) the masking of fever can delay antibiotic treatments, ii) fever is protective *per se* and iii) NSAIDs decrease the production of molecules that are involved in the recovery [12–14]. Indeed, prostaglandin E2 (PGE2) and prostacyclin (PGI2), the production of which are inhibited by NSAID, protect against free radicals, suppress the inflammatory cell recruitment in the lung and induce tissue repairing processes in rats and human [15–18]. Therefore, NSAIDs might augment the neutrophil influx, activity and life-span.

The inflammatory response plays an important role in the pathogenesis of BRSV, similarly to respiratory bacterial infections. The virus induces an excessive neutrophil response, characterised by extracellular DNA, mucus and neutrophil secretory proteins that generate lung fibre destruction and airway plugs (neutrophil extracellular traps, NETs) [19, 20]. This study aimed to determine if the use of NSAID in the early phase of BRSV-infection is beneficial in calves and if it limits pulmonary inflammation.

Effects of two NSAIDs were investigated in 4-to-9-week old calves that were infected with BRSV by aerosol: meloxicam (Metacam®), which at least in humans preferentially inhibits COX-2 [21], and DL-lysine acetylsalicylate (Aspirin®), which acetylates COX-1 to a larger extent than COX-2 [21, 22]. The aspirin-mediated acetylation is irreversible and inhibits the function of COX-1, however in COX-2, it may induce a switch to the generation of aspirin-triggered specialised proresolving mediators (SPM) with anti-inflammatory and healing properties [23]. Effects on clinical signs, viral replication and lung lesions, as well as proteomic and lipidomic profiles in BAL and plasma were evaluated, to characterise the effects of meloxicam and DL-lysine acetylsalicylate (hereafter called aspirin) on the inflammation induced by BRSV.

## Materials and methods

### Challenge virus

Foetal bovine turbinate cells (CRL-1390™, ATCC, UK), propagated as described previously [24], were used for virus isolation and culture. The cells were free from bovine viral diarrhoea virus as determined by immunostaining using polyclonal antisera (PA0042, VLA, UK).

The challenge virus consisted of a BRSV field strain (GenBank accession number MG947594) that belonged to genotype subgroup II [25, 26]. This virus had been isolated from the nasal secretions of a 3-month-old dairy calf during an outbreak of respiratory disease (BRSV/Sweden/HPIG-SLU-620-Lovsta/2016) and had been passed six times in cell culture and twice in calves. The virus was free from nucleic acid coding for *Mycoplasma bovis*, *Mannheimia haemolytica*, *Pasteuella multocida*, *Hemophilus somni*, bovine coronavirus and bovine parainfluenza virus type 3, as determined by RT-PCR, LSI VetMaxTM Screening Pack, Ruminant Respiratory Pathogens (Life technologies, France, and DNA /RNA extraction using the DNeasy Blood & Tissue Kit and the RNAeasy® Mini kit (Qiagen, Sweden) respectively, according to the manufacturers’ instructions. Sweden is free from bovine herpes virus type 1, bovine leucosis virus and bovine viral diarrhoea virus since 1995, 2001 and 2014, respectively.

### Animals and experimental design

**Fig 1:**
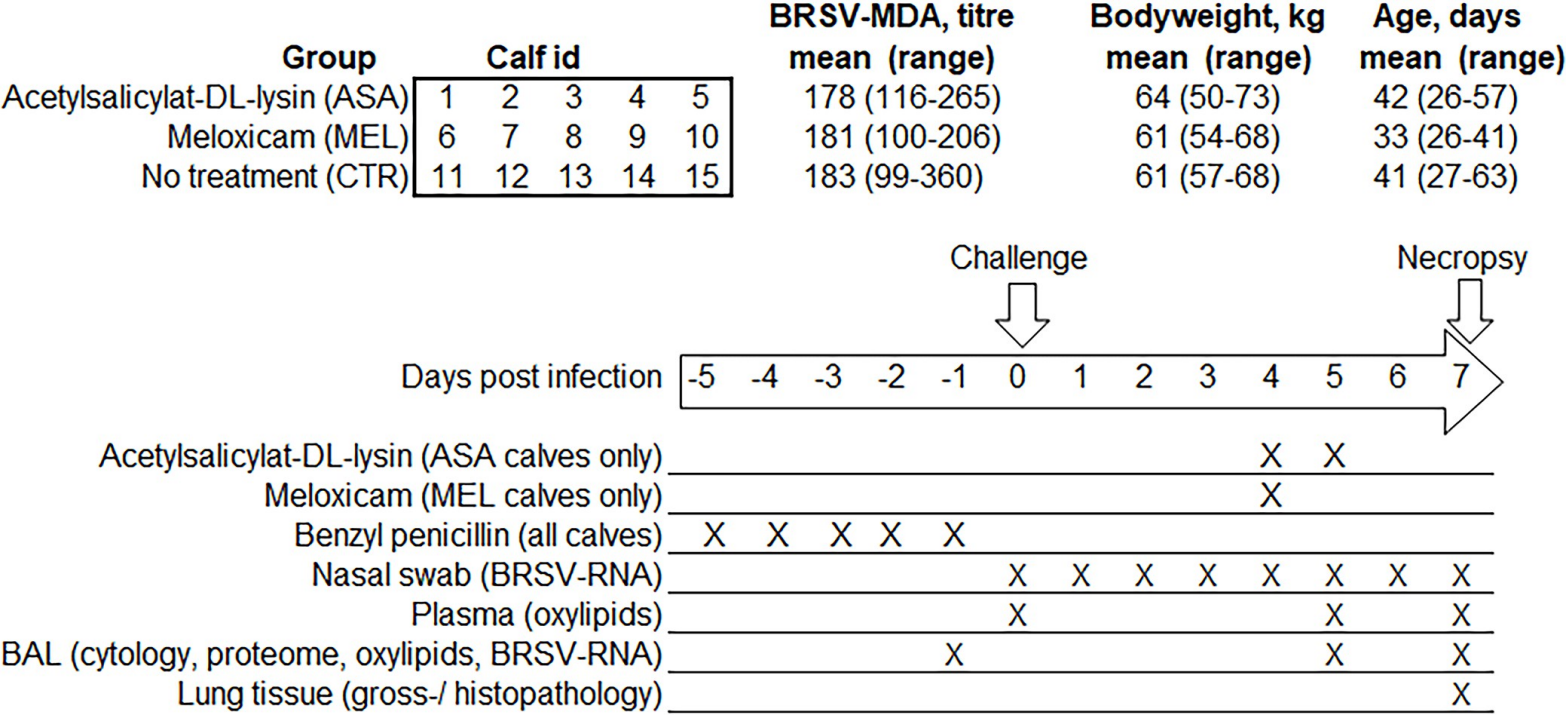
Experiment design. Calves were infected with BRSV by aerosol on day (D)0 and were treated with either meloxicam intravenously on D4 (n = 5, MEL), acetylsalicylat-DL-lysin intravenously on D4 and D5 (n = 5, ASA), or were left untreated (n = 5, CTR). Clinical signs were monitored daily until necropsy on D7. Samplings and analyses were performed as indicated.

Fifteen 4-to-9-week-old healthy female dairy calves (Swedish red and white and Swedish Holstein breeds) without history of disease were transported from Lövsta research farm of SLU, Uppsala, Sweden, to the Swedish Veterinary Agency, Uppsala, Sweden. An outbreak of BRSV had occurred in the herd 19 months earlier and BRSV infections had then been ruled out by serological monitoring of young stock in the herd, as previously described [27]. Based on serology on milk of first parity cows performed within a voluntary surveillance programme, the herd was considered free from *Streptococcus agalactie*, *Salmonella* and *Mycoplasma bovis*. Calves were allocated according to age, bodyweight (BW) and BRSV-specific maternally derived antibodies (MDA) to three treatment groups and five group pens that contained one calf per treatment group (Fig 1).

Between D-5 and D-1, all calves were treated intramuscularly with 40 mg procaine benzylpenicillin per kg BW. On D0, all calves were challenged with 10^4^ TCID_50_ BRSV by aerosol, as described previously (Blodörn et al 2013) [33]. Treatments were randomly allocated to the treatment groups. Calves received either 0.5 mg/kg meloxicam (MEL, Metacam® for cattle, swine and horse, 20 mg/ml, Boehringer Ingelheim Animal Health, Denmark) intravenously on D4 post-infection (n = 5), 20 mg/kg acetylsalicylat-DL-lysin (ASA, Aspirin® i.v., 100 mg/ml, Bayer, Germany) intravenously on D4 and D5 (n = 5), or no treatment (n = 5, CRT). The calf allocation and experiment layout are illustrated in Fig 1.

### Monitoring of clinical signs of disease and sampling *in vivo*

Clinical signs of disease were monitored and scored on a daily basis, as described (Blodörn 2013). Nasal swabs (UTM® Copan, Italy) were collected from D0 to D7 and bronchoalveolar lavages (BAL) were performed D-1 and D5, as described (Valarcher et al 2021). Four ml of peripheral blood was collected from the jugular vein on D0, D5 and D7, in tubes containing 7.2 mg EDTA (BD Vacutainer®).

### Preparation of plasma and BAL collected *in vivo*

The blood was centrifuged within 10 minutes of sampling at 3000 x*g* for 10 min and 4°C and the resulting plasma was immediately frozen on dry ice, before storage at -75°C until oxylipid analysis. The BALs were immediately filtered through sterile gauze and aliquoted. For each BAL, one aliquot was frozen on dry ice and then stored at -75°C until oxylipid analysis, one was prepared using cytospin for cytological evaluation, one received glycerol (G5516, Sigma-Aldrich) to a final concentration of 15% and was stored at -75°C until bacterial culture. Other aliquots of filtered BAL were centrifuged at 200 x*g* for 10 min and 4°C and the supernatant was removed and stored at -75°C until analysed by mass spectrometry-based proteomics. The cell pellets were resuspended in 500 μl PBS and stored at -75°C until BRSV-RNA extraction and RT-qPCR analysis.

### Post mortem sampling and histology

Post mortem BALs were performed as described previously [28] and prepared as above and the extent of macroscopic lung lesions were drawn on a lung chart and quantified by Image J (free software, version 1.52a). Lung tissue was obtained from the cranial lobe, preferentially from areas with lesions, and was stored in 10% neutral buffered formalin solution for 24 hours until dehydration with ethanol and paraffin embedding. Histopathological analyses were performed on 5 μm thick sections. The sections were stained with hematoxylin and eosin, the inflammation was characterised and the presence of neutrophils (intraepithelial and in airways, *i*.*e*. in bronchial, bronchiolar, and alveolar lumen) was scored between 0 and 3 by a pathologist (EL) who was blind to treatment groups. An average of the two scores was calculated and expressed as lung histology neutrophil score.

### Detection of virus and bacteria

Ribonucleic acid was extracted from 200 μl nasal swab medium or BAL cells in PBS using an automated nucleic acid platform (Maelstrom™ 9600, TAN-BEAD, Taiwan) and the IndiMag Pathogen kit (SP947257, Indical Bioscience, Germany). Virus RNA was detected by RT-qPCR by using BIO-T KIT® BRSV & PI3 (Biosellal, France), according to the manufacturer’s instructions. The unit TCID_50_ equivalent (TCID_50_ eq.) was used as the standard curve used in the assay was based on a BRSV-infected cell lysate with a known titer. Bacterial culture was performed on BAL in the routine diagnostic in a blind manner at the Swedish National Veterinary Agency according to SS-EN ISO/IEC 17025, by enrichment, aerobic culture and typing by matrix-assisted laser desorption/ionisation.

### Cytology

The BALs were processed at Clinical Pathology Laboratory, University Animal Hospital, Swedish University of Agricultural Sciences within 2 hours of collection. The total nucleated cell count was determined using Advia 2120 (Siemens Healthcare GmbH, Ashburn, Germany). Cytospins were performed by using 100 μl BAL fluid or resuspended BAL cells (prepared by centrifuging 10 ml BAL at 500 x *g* for 5 min) diluted 1:5 in albumin solution (1 g bovine serum albumin and 0.002 g NaN3 dissolved in 10 ml of 0.9% NaCl) and loaded in cytocentrifuge cassettes (Thermo Scientific Cytospin 4 centrifuge, Thermo Fisher Scientific, Waltham, Massachusetts, US). Slides were stained with May-Grünwald-Giemsa and evaluated by a clinical pathologist (AH), who was blinded for group assignment and other information about the calves. A 400 differential count was performed and cells were classified as macrophages, lymphocytes, neutrophils, mast cells or eosinophils, expressed as a percentage. Results from calf 2, 3, 5, 10 and 14 obtained from samples collected on D-1 were discarded because the collection of BAL was not successful.

### Label-free quantitative mass spectrometry-based proteomics

Proteins in BAL supernatants (in a volume corresponding to 7 μg of total protein) were identified and semi-quantified by liquid chromatography and tandem mass spectrometry (LC-MS/MS), as previously described [29]. Briefly, after reduction, alkylation, in-solution digestion by trypsin, purification by Pierce C18 Spin Columns (Thermo Scientific) and drying, the peptides were resolved in 0.1% formic acid. The peptides were separated in reversed-phase by using an EASY-nLC 1000 system, a C18 Spin Column (Thermo Scientific, Germany) and a 90 min long gradient, and were electrosprayed online to a Q Exactive Plus Orbitrap mass spectrometer (Thermo Finnigan, Germany). Tandem mass spectrometry was performed applying higher-energy collisional dissociation fragmentation. All samples were analysed on the same column to allow comparisons. Database searches were performed in the MaxQuant software (version 1.5.3.30). Proteins were identified by searching against the database Bos taurus proteome, extracted from Uniprot 2021-01-13. Fixed modification was carbamidomethyl, and variable modifications were oxidation and deamidation. A decoy search database, including common contaminants and a reverse database, was used to estimate the identification false discovery rate. The RAW-data files were quantitatively analysed by the quantification software MaxQuant 1.5.3.30 and the results of all fractions were combined to a total label free intensity analysis for each sample.

The label-free protein-quantities (LFQ) in BAL from D5 and D7 were analysed according to Aguilan *et al*. (2020) [30]. Briefly, the data was first filtered by including only proteins that were identified in at least four calves within at least one out of two groups compared. The data was then transformed by log2 and normalised by scaling each value against the average of all proteins in a given sample. The normalisation aimed to correct for artificial biases and to allow comparison of relative abundance of individual proteins, since the total protein content for the conditions compared was the same. To be able to calculate fold changes, the probabilistic minimum imputation method was used, in which empty cells were replaced with artificial distribution of values close to the limit of detection but with a variability comparable to the detected values [30]. Pathway analysis was performed by using the software Ingenuity Pathway Analysis (version 90348151, 2023 QIAGEN).

### Lipidomics

Oxylipids were detected in BAL and plasma by using liquid chromatography/tandem mass spectrometry (LC/MS/MS) with triple quadrupole mass detectors.

Initial analyses were performed within the Metabolomics unit, SciLifeLab Infrastructure, Sweden, by using an ultra-performance LC system (Agilent Infinity 1290) coupled with an electrospray ionisation source to a triple quadrupole mass detector (Agilent 6495, Agilent Technologies, Santa Clara, CA, USA) equipped with iFunnel Technology, as previously described [31]. Because the set of BALs obtained on D-1 was not complete, and due problems with the metabolite separation in BAL from D7 using columns (Waters BEH C18), only results for plasma (D0, D5 and D7) and for BAL collected on D5 were considered valid.

Additional analyses were thereafter performed on BAL collected on D5 and D7, by Ambiotis, France, according to methods described previously [32]. Briefly, after protein precipitation with methanol, the samples were extracted by solid-phase extraction using oasis HLB 96-wells plates (Waters, Europe) and lipids mediators were eluted with methanol and methyl formate.

### Statistical methods

Analyses of the BAL proteomic data were performed in Excel, Microsoft Office 2016 with a plugin available at http://www.real-statistics.com. The Shapiro-Wilk normality test was used, followed by the Mann-Whitney test for non-normally distributed data and the two sample T test for normally distributed data. The F-test was used to check if the data was homosedastic or heterosedastic. Criteria used for differential expression were p-values <0.05 (-log2 p-value >4.3219) and > 2 fold change (>1 log2 fold change) [30]. This method was chosen to reveal patterns of changes induced by treatment on the BAL proteome of BRSV-infected calves, for further analysis of biological pathways by IPA and consequent creation of new hypotheses to be verified in further studies. The remaining data were analysed in Minitab (version 16) by using the mixed effects model for repeated measures with the restricted maximum likelihood estimation method and Kenward-Roger approximation for fixed effects. Calf identity was used as random factor and day as well as treatment were used as fixed factors. Data that were not normally distributed were log transformed and Tukeys’ test with 95% confidence was used to correct for multiple testing.

### Ethics

Approval was obtained from the Ethical Committee of the district court of Uppsala, Sweden (Ref. no. 5.8.18–13628_2021). The calves were housed in a biosafety level 2 facility, in group pens on a thick layer of sawdust to have a good lying comfort. They were frequently and gently handled to reduce stress during injections and samplings. Bronchoalveolar lavage were performed under local anesthesia (lidocaine 2%, gel and spray). Hay and water were given *ad libitum* and the calves received 6 litres of milk replacer (Delikat, Svenska Foder AB, Lidköping, containing 2750 ppm alpha-linolenic acid per kg) per day. Sacrifice was performed by an overdose of general anesthesia administered intravenously (0.1 mg/kg BW xylazine, 15 mg/kg BW ketamine and 30 mg/kg BW pentobarbital), followed by exsanguination.

## Results

### Neither aspirin, nor meloxicam improved clinical signs induced by BRSV

**Fig 2:**
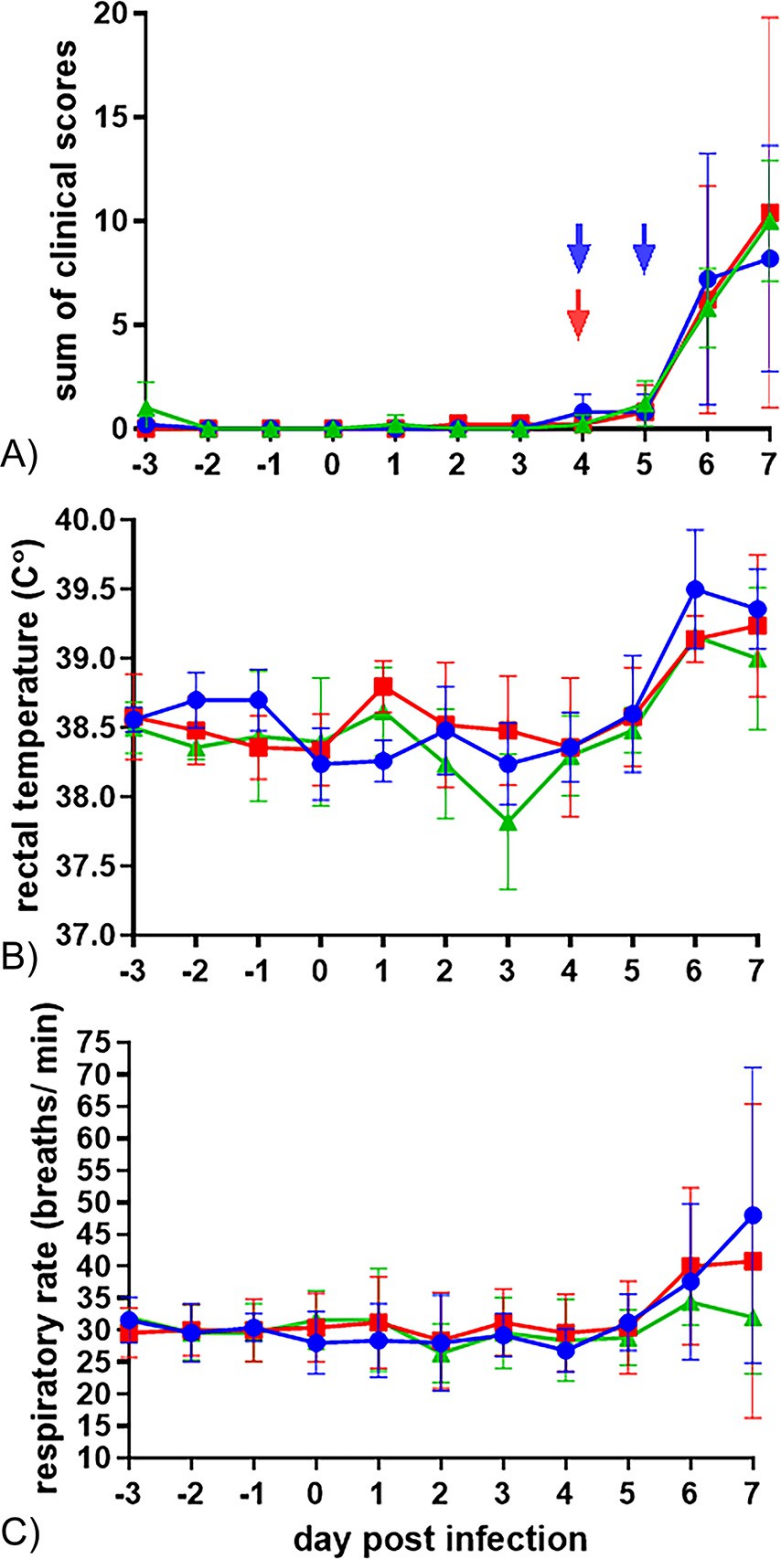
Lack of effect of early treatment with NSAIDs on clinical parameters following BRSV-infection of calves. Mean (± SD) (A) clinical scores (B) rectal temperatures and (C) respiratory rates of calves infected with BRSV by aerosol on day (D)0 and treated with either meloxicam intravenously on D4 (n = 5, MEL), acetylsalicylat-DL-lysin intravenously on D4 and D5 (n = 5, ASA), or untreated (n = 5, CTR).

The calves developed mild to moderate clinical signs of respiratory disease including increased rectal temperature, tachypnea, wheezing on lung auscultation and serous to mucopurulent nasal discharge. No significant differences were observed between groups with regard to clinical score, rectal temperature or respiratory rate (Fig 2).

**Fig 3:**
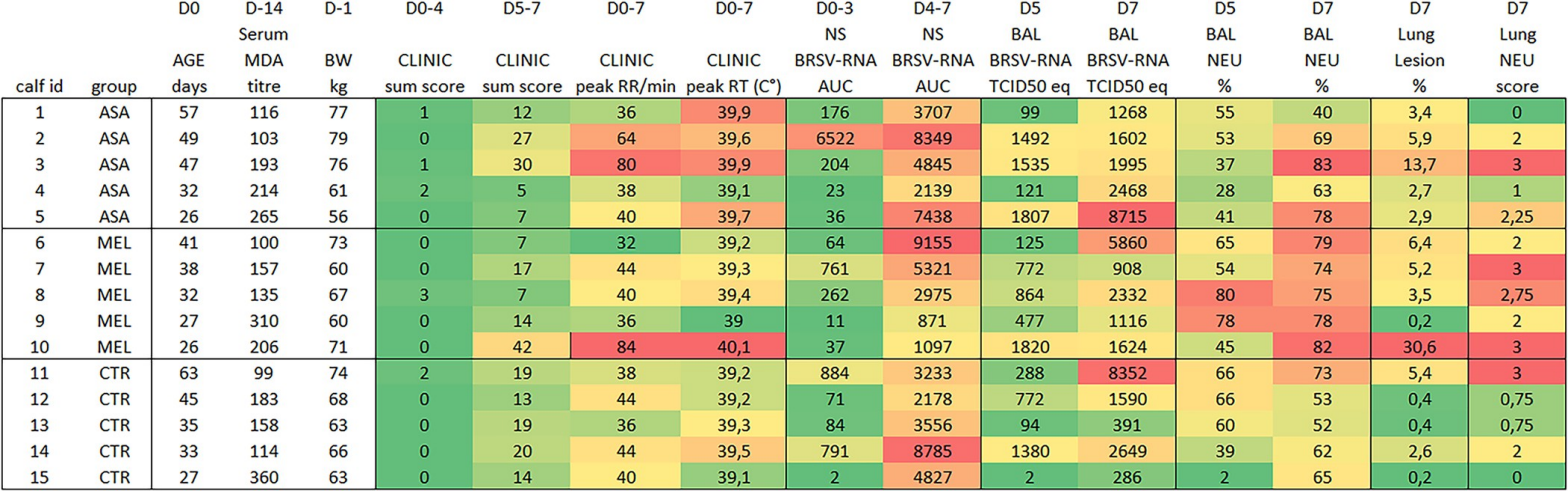
Individual clinical, virological, pathological and inflammatory outcomes following BRSV-infection and NSAID-treatment of calves. Calves were infected with BRSV by aerosol on day (D)0 and were treated with either meloxicam intravenously on D4 (n = 5, MEL), acetylsalicylat-DL-lysin intravenously on D4 and D5 (n = 5, ASA), or were left untreated (n = 5, CTR). Clinical signs were monitored and scored daily until necropsy on D7. BRSV-RNA was detected in nasal swabs (NS) and bronchoalveolar lavage (BAL) by RT-qPCR, macroscopic lesions and neutrophils were quantified in BAL and lung tissue. MDA; maternally derived antibodies, RR; respiratory rate, RT; rectal temperature, AUC; area under the curve, NEU; neutrophil.

The calves with highest peak respiratory rate and sum of clinical scores D5-D7 (calf 3 and 10, Fig 3) and those with highest peak rectal temperature (calf 5 and 10) were among those treated with either meloxicam (calf 10) or aspirin (calf 3 and 5).

### Neither aspirin, nor meloxicam significantly affected BRSV replication

**Fig 4:**
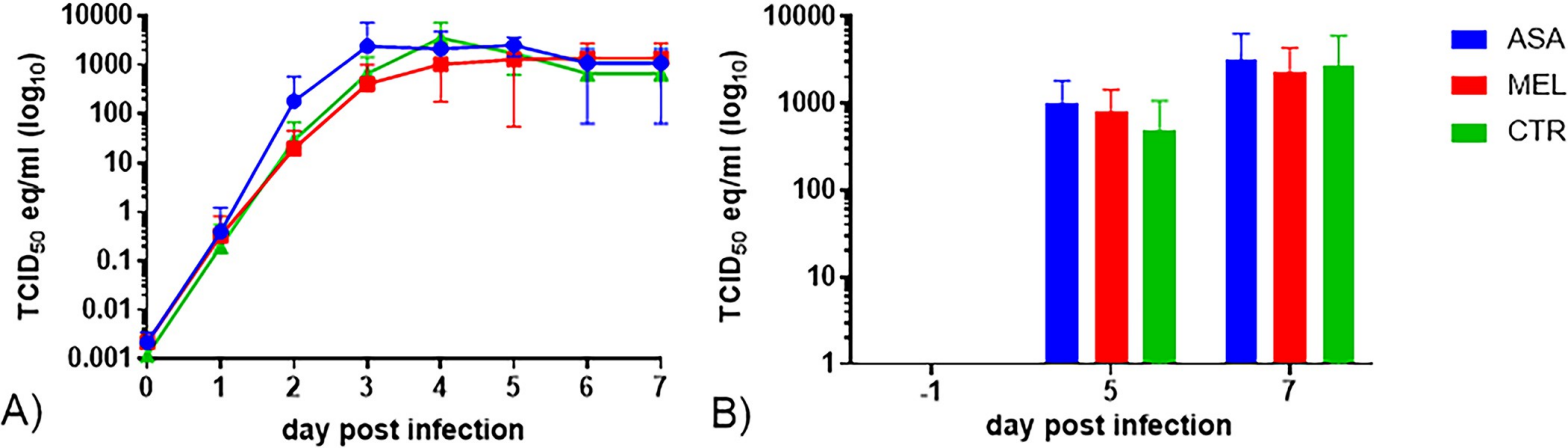
Lack of effect of early treatment with NSAIDs on BRSV replication in calves. Mean (± SD) (A) 50% Tissue Culture Infection Dose equivalent units (TCID_50_ eq), estimated from BRSV-RNA quantity detected by RT-qPCR in nasal swabs and (B) broncho alveolar lavage (BAL) calves infected with BRSV by aerosol on day (D)0 and treated with either meloxicam intravenously on D4 (n = 5, MEL), acetylsalicylat-DL-lysin intravenously on D4 and D5 (n = 5, ASA), or untreated (n = 5, CTR).

All calves shed virus from D2 or D3 throughout D7 and there were no significant differences in the quantity of BRSV-RNA detected, neither in nasal swabs, nor in BAL, between calves in different treatment groups (Fig 4A and 4B, respectively).

Among meloxicam-treated calves and controls, those with the lowest BRSV-specific maternally derived antibody (MDA) titer at challenge had the highest quantity of BRSV-RNA in BAL on D7 (calf no. 6 and 11, Fig 3), but this was not the case among aspirin-treated calves (calf no. 2, Fig 3).

The calves with highest peak respiratory rate and sum of clinical scores D5-D7 (calf 3 and 10, Fig 3), did not have the highest quantity of BRSV-RNA, neither in nasal secretions D4-7, nor in BAL D7 (Fig 3). However, calf 10 had the highest quantity of BRSV-RNA in BAL on D5 (Fig 3), despite that it had high BRSV-specific MDA titers at challenge.

### Clinical signs and lesions were not associated with bacterial infection

No bacterial respiratory pathogen was detected in the BAL of any calf at D7. Furthermore, no bacterium was detected in BALs from calves with peak respiratory rate and/or rectal temperatures (calves no. 3, 5 and 10, Fig 3 and S1 Table), or in those with highest proportion of BAL neutrophils and lung lesions (calves no. 3 and 10, Fig 3 and S1 Table). However, some of the BALs at D7 contained sparse bacteria that were detected by culture (in 1/5 aspirin treated calves; in 4/5 meloxicam-treated calves and in 3/5 controls, S1 Table), but not by cytology.

### Meloxicam-treated calves had increased neutrophil influx in BAL

**Fig 5:**
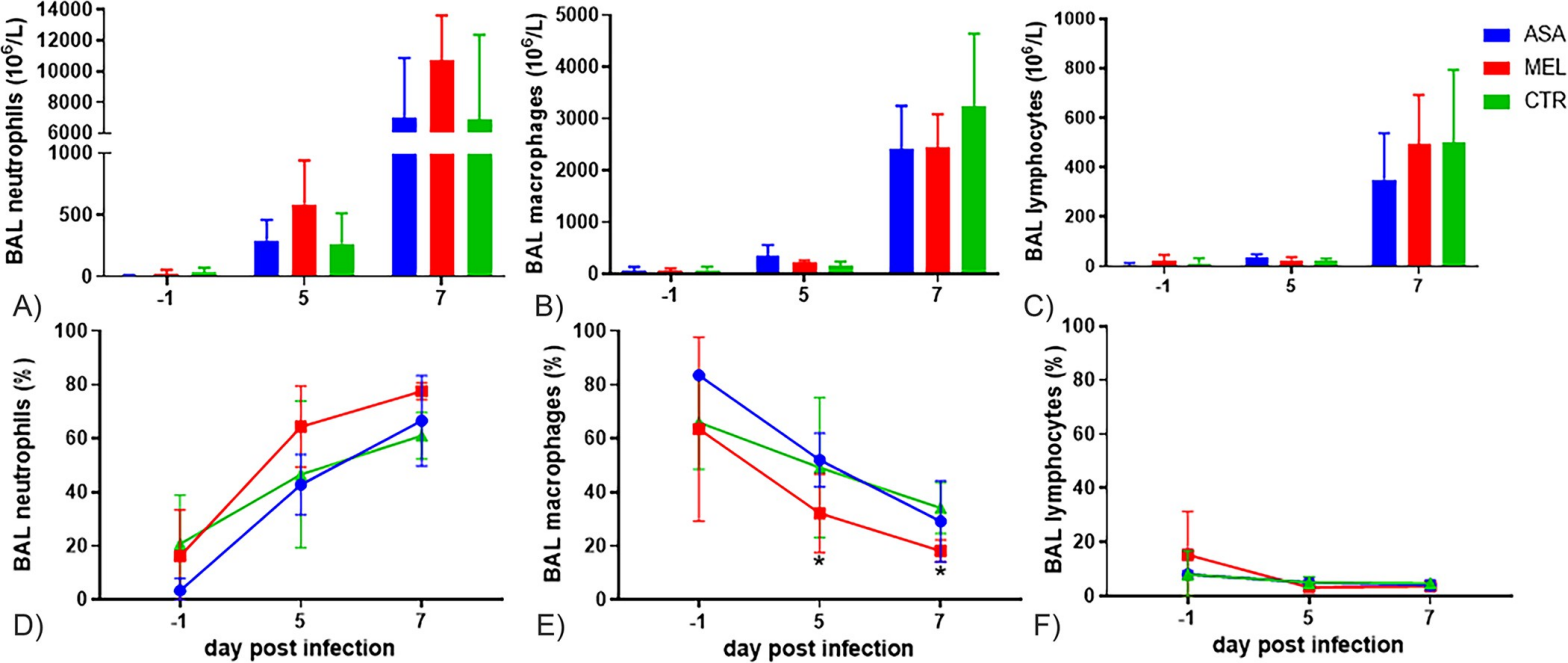
White cells in bronchoalveolar lavage of BRSV-infected calves. Mean concentration (± SD) of (A) neutrophils (B) macrophages and (C) lymphocytes, or mean proportion (± SD) of (D) neutrophils (E) macrophages and (F) lymphocytes in bronchoalveolar lavage (BAL) of calves infected with BRSV by aerosol on day (D)0 and treated with either meloxicam intravenously on D4 (n = 5, MEL), acetylsalicylat-DL-lysin intravenously on D4 and D5 (n = 5, ASA), or untreated (n = 5, CTR). For significance, please see text.

BRSV induced an influx of inflammatory cells in the airway lumen, which consisted mainly of neutrophils, but also of macrophages and, to a lesser extent, of lymphocytes (Fig 5A–5C, please note that the scales of the Y-axes differ).

The proportion of neutrophils in BALs successively increased, whereas the proportion of macrophages and lymphocytes decreased (Fig 5D–5F). By comparing two groups at a time, day by day in a two sample T-test, Meloxicam-treated calves had significantly higher proportion of neutrophils and lower proportion of macrophages in BAL compared to aspirin-treated calves on D5 (p = 0.037 and p = 0.041, respectively) and compared to untreated controls on D7 (p = 0.01 and p = 0.018, respectively). Furthermore, in the mixed model effect analysis for repeated measures, which included all groups and multiple samplings, the effect of treatment on the proportion of neutrophils and macrophages was significant (p = 0.036 and p = 0.035, respectively).

By additionally correcting for multiple comparisons in Tukeys test, meloxicam-treated calves D7 were the only with a significantly higher proportion of neutrophils than all groups of calves D-1 and than aspirin-treated calves and controls on D5. Conversely, meloxicam-treated calves D7 were the only with a significantly lower proportion of macrophages than all calves D-1, and than aspirin-treated calves and controls on D5. By this method, although the effect of treatment on the proportion of neutrophils was significant, the differences between treatment groups on the same day were not significant.

### Macroscopic lesions were limited in most calves

**Fig 6:**
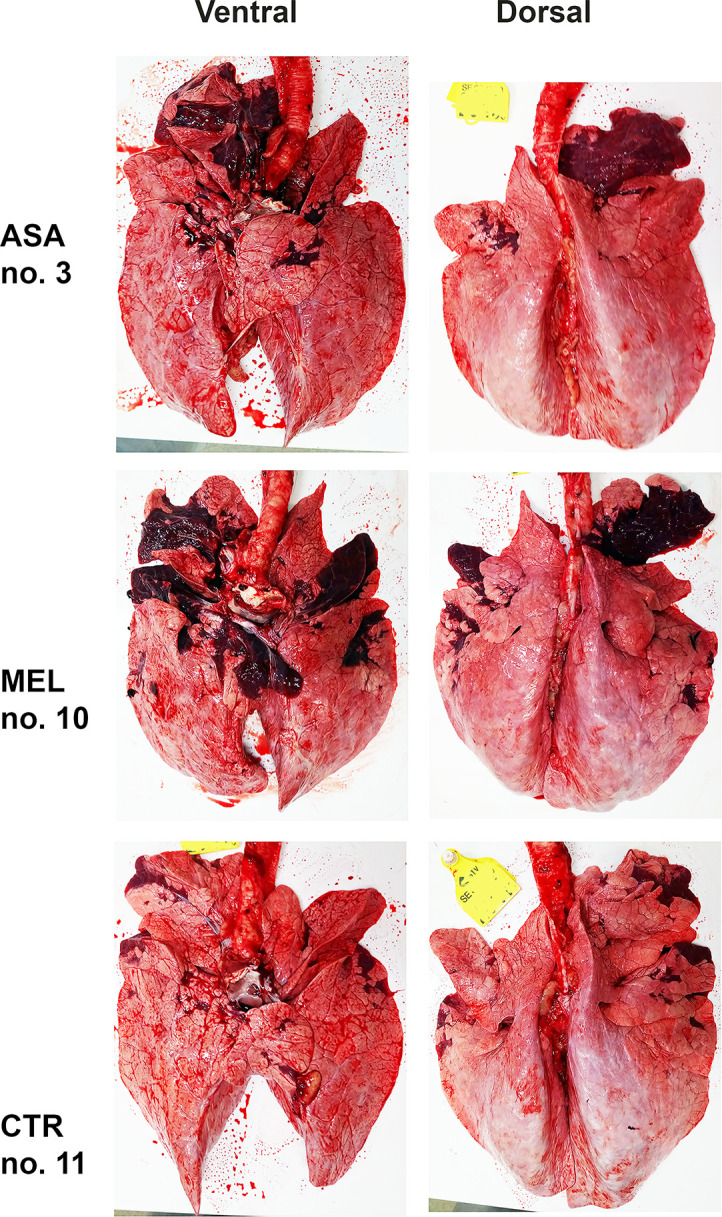
Lung lesions in BRSV-infected, meloxicam- or asprin-treated calves and untreated controls. Photographs of lung lesions D7 post infection, represented by the individual with the most extensive lesion within its group (please see Fig 3). The brightness was adjusted in the same manner for all photographs. Calves were infected with BRSV on day D0 and treated with either meloxicam intravenously on D4 (MEL), acetylsalicylat-DL-lysin intravenously on D4 and D5 (ASA), or untreated (CTR).

Overall, the gross pathological lung lesions were limited, except in one calf treated with aspirin and in one calf treated with meloxicam (no. 3 and 10, Figs 3 and 6), in which broncho-interstitial pneumonia was observed.

Overall, gross pathologic lesions included a slightly collapsed parenchyma and densification of parts of cranial and medial pulmonary lobes, along with mucopus in the bronchus and bronchi. The mean (range) extent of lesions was 6% (3% - 14%), 9% (0.2% - 31%) and 2% (0.2% - 5%) for aspirin-treated calves, meloxicam-treated calves and controls, respectively, but this difference was not significant (Figs 3 and 6).

### Neither aspirin, nor meloxicam reduced the number of neutrophils in lung tissue

According to histopathological analyses, the pulmonary inflammation was characterized as interstitial in calf 1 (aspirin-treated), 4 (aspirin-treated), 12 (control), 13 (control) and 15 (control), and as bronchointerstitial in the rest of the calves. The histopathological neutrophil scores did not differ significantly between groups, but the average score was highest in meloxicam-treated calves. The two calves with the most extensive lung lesions (calf 3 and 10) had the highest overall neutrophil score, together with one control with high quantities of BRSV-RNA in BAL on D7 (calf 11) and one meloxicam-treated calf (calf 7, Fig 3).

### Both aspirin and meloxicam affected the protein expression in the airways

A large number of proteins was identified in BAL by Label-free Quantitative Mass Spectrometry-Based Proteomics (268–939 proteins per calf and sampling occasion).

**Fig 7:**
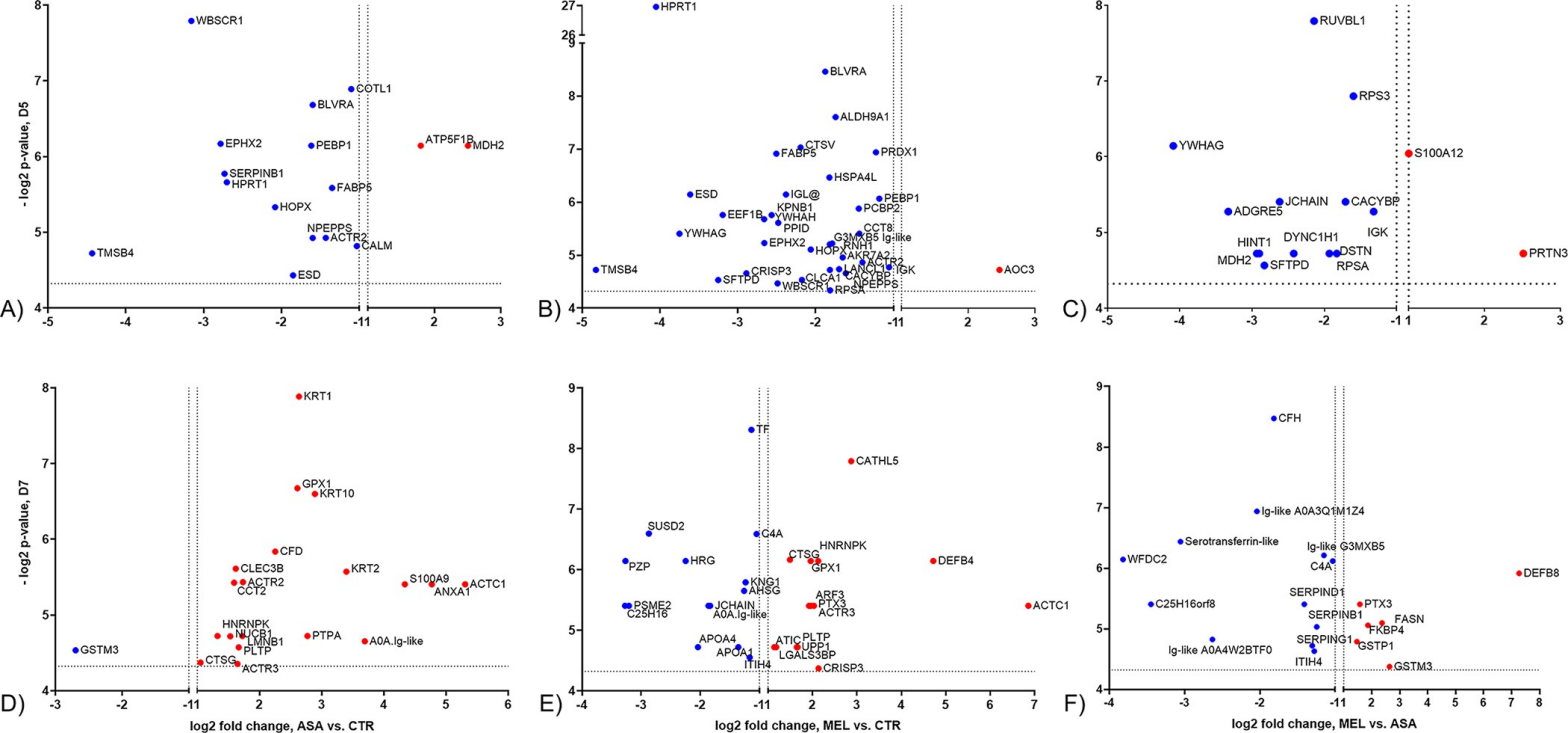
Differentially expressed proteins in bronchoalveolar lavage of BRSV-infected, aspirin- or meloxicam-treated calves and controls. Proteins detected by label-free quantitative mass spectrometry-based proteomics, with differential expression in bronchoalveolar lavage (BAL) collected on day (D)5 post infection, between (A) aspirin-treated calves and controls (B) meloxicam-treated calves and controls and (C) aspirin-treated and meloxicam-treated calves. Proteins with significantly differential expression in BAL collected on D7 post infection, between (D) aspirin-treated calves and controls (E) meloxicam-treated calves and controls and (F) aspirin-treated and meloxicam-treated calves. The p-value is expressed in log2 and the fold-change is based on log2-transformed, normalised, label-free quantitation units, after imputation. Dots in red and blue colour represent proteins that are upregulated and downregulated, respectively, with regard to the former group relative to the latter group, which are named on the X-axis. For example, TMSB4 is downregulated in aspirin-treated calves relative to controls on D5 (A).

Overall, only a few proteins differed significantly and more than two-fold between groups (*i*.*e*. were considered as differentially expressed). On D5 (24 h post treatment), a majority of these proteins were downregulated in both aspirin- and meloxicam-treated calves relative to controls (14/16 for ASA and 33/34 for MEL, Fig 7A and 7B). In addition, meloxicam mainly downregulated proteins relative to aspirin (Fig 7C).

On D7, proteins were either mainly upregulated in treated calves (aspirin, 19/20, Fig 7D) or were both up and downregulated (meloxicam, 14 up, 14 down, Fig 7E), compared to controls. Meloxicam downregulated the majority of the differentially expressed proteins relative to aspirin (Fig 7F).

**Fig 8:**
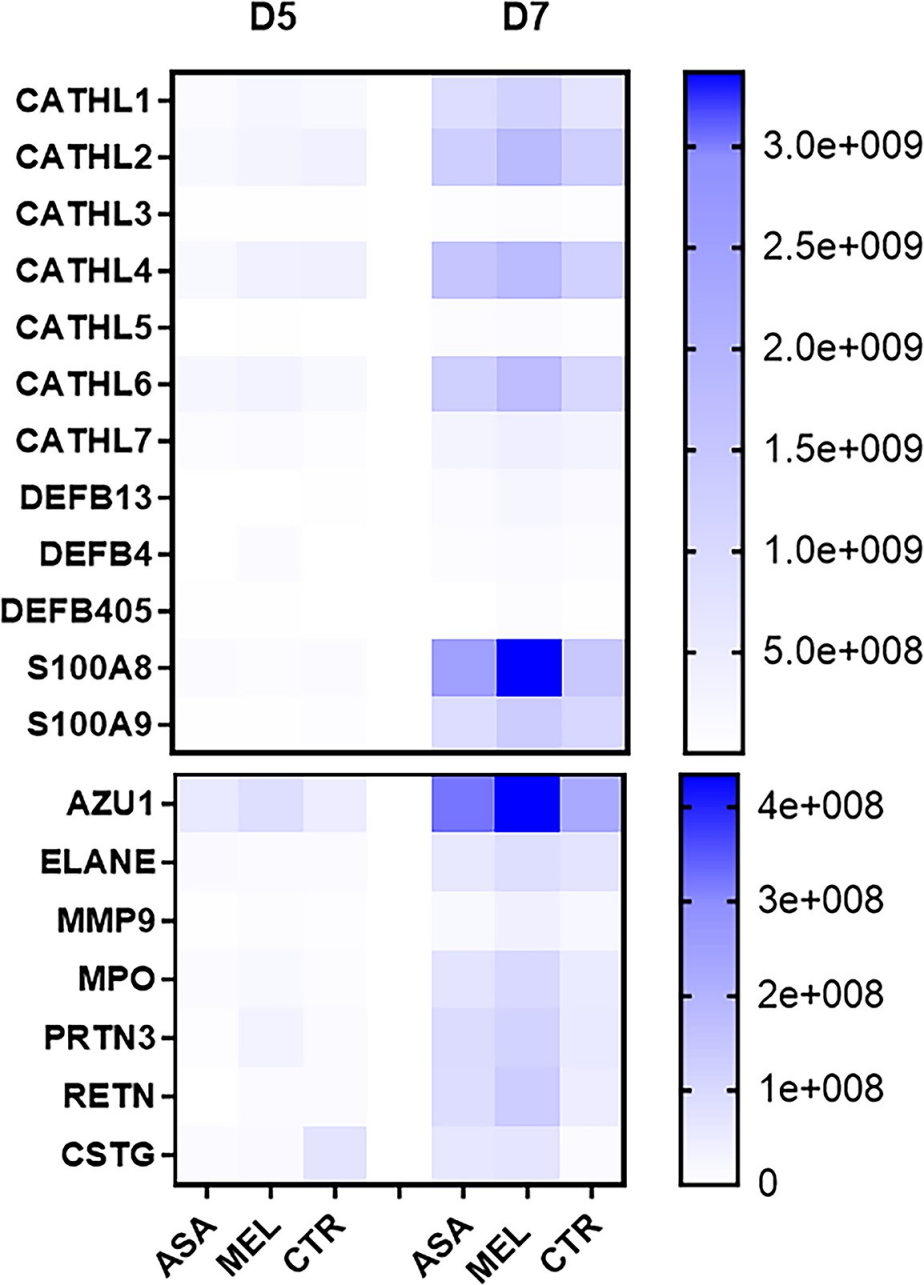
Neutrophil-related proteins in bronchoalveolar lavage of BRSV-infected, aspirin- or meloxicam-treated calves and controls. Mean relative quantity of neutrophil-related proteins detected by label-free quantitative mass spectrometry-based proteomics in bronchoalveolar lavage (BAL) collected on day (D)5 and D7 post infection.

In the total filtered BAL proteome, 19 neutrophil-related proteins were identified and quantified. The average relative quantities of these proteins were consistently higher on D7 than on D5 and were on average highest in meloxicam-treated calves (Fig 8). By using the mixed model effect analysis for repeated measures, corrected for multiple comparisons, meloxicam-treated calves had significantly higher proportion of cathelicidin 5 (CATHL5, p = 0.045) and tended to have higher proportion of azurocidin (AZU1, p = 0.076) compared to controls. At 91% (but not 95%) confidence, meloxicam-treated calves had significantly higher relative quantity of AZU1 compared to controls on D7 and compared to all groups on D5.

Overall, proteins that were related to immunoglobulins, several of which were unmapped (*i*.*e*. not recognised) by the IPA software, were downregulated in the BAL of meloxicam-treated calves. The immunoglobulin kappa and lambda lokus (IGK and IGL@) and the Ig-like domain-containing protein G3MXB5 were significantly downregulated in meloxicam-treated calves relative to untreated controls on D5 (Fig 7B). The meloxicam-treated calf 10 (the calf with the most extensive lung lesions) was the only calf in which IGL@ was not detected on D5. Similarly, relative to aspirin-treated calves, there was a downregulation or consumption of the IGK and JCHAIN in meloxicam-treated calves on D5 (Fig 7C).

On D7, the joining chain of multimeric IgA and IgM (JCHAIN) and the Ig-like domain-containing protein A0A3Q1M1Z4 were downregulated in meloxicam-treated calves relative to untreated controls (Fig 7E). Moreover, the Ig-like domain-containing proteins A0A3Q1M1Z4, A0A4W2BTF0 and G3MXB5, serpin B1, D1 and G1 and complement C4A were downregulated in meloxicam-treated calves relative to aspirin-treated calves (Fig 7F). The complete list of differently regulated proteins is presented in S2 Table.

On D5, no Ingenuity canonical (metabolic and cell-signaling) pathway that included more than three proteins was identified as significant in aspirin- or meloxicam-treated calves compared to controls.

In the comparison between aspirin-treated relative to meloxicam-treated calves, two such pathways were identified (S100 family signaling pathway and neutrophil degranulation, with 4/5 and 2/4 proteins downregulated in meloxicam-treated calves, p = 7.6x10^-5^ and p = 1.7x10^-4^).

On D7, no Ingenuity canonical pathway that included more than three proteins was identified as significant in aspirin-treated calves relative to controls.

For meloxicam-treated calves relative to controls on D7, the top three Ingenuity canonical pathways with highest significance and that contained more than three proteins were:

- LXR/RXR Activation (7/8 proteins downregulated, p = 4.14x10^-13^)
- FXR/RXR Activation (7/8 proteins downregulated, p = 5.04x10^-13^)
- DHCR24 Signaling Pathway (7/8 proteins downregulated, p = 9.95x10^-13^)

For meloxicam-treated calves relative to aspirin-treated calves on D7, only one significant pathway was identified that contained more than three proteins, namely acute phase response signaling (4/4 proteins downregulated in meloxicam-treated calves, p = 1.6 x10^-6^).

### The quantity of BRSV-RNA D5 and neutrophil-related proteins D7 in BAL correlated with the extent of lung lesions

Correlation analyses were performed including all calves in the same analysis between the extent of lesions and i) BRSV-RNA in nasal secretions and BAL on D5 and D7, ii) neutrophils in BAL on D5 and D7, and iii) neutrophil- or immunoglobulin-related proteins detected by label-free quantitative mass spectrometry-based proteomics. The extent of lung lesions tended to have a linear association with the proportion of neutrophils in BAL on D7 and a significant association was detected between lesions and BRSV-RNA in BAL D5, as well as with the relative quantity of 10/18 neutrophil-related proteins in BAL D7 (Table 1). In contrast, this association was not identified for immunoglobulin-related proteins. The calf with the most extensive lung lesions (no. 10, meloxicam-treated) had the highest quantity of BRSV-RNA in BAL on D5 and the highest relative quantity of AZU1, ELANE and MMP9 in BAL on D7 among all calves. In contrast to calf 10, which had relatively low quantities of BRSV-RNA in BAL on D7, the control calf with the most extensive (although limited) lung lesions within its group (no. 11) had the highest levels of BRSV-RNA within its group in BAL on D7, as is usually observed within this model [24, 33].

**Table 1:** Linear association between lung lesions of BRSV-infected meloxicam-or aspirin-treated calves and untreated controls and BRSV-RNA, neutrophils and neutrophil-related proteins in airways.

		R	p-value
**Neutrophil-related** **proteins in BAL D7**	CATHL1	0.76	**
	CATHL2	0.47	ns (0.08)
	CATHL3	0.61	*
	CATHL4	0.60	*
	CATHL5	0.17	ns
	CATHL6	0.70	**
	CATHL7	-0.03	ns
	DEFB13	0.70	*
	DEFB4	0.50	ns
	DEFB405	0.68	ns
	S100A12	-0.13	ns
	S100A8	-0.01	ns
	S100A9	0.07	ns
	AZU1	0.65	**
	ELANE	0.75	**
	MMP9	0.80	**
	MPO	0.56	**
	PRTN3	0.32	ns
	RETN	0.72	**
**BRSV RNA**	nose D5	0.01	ns
	BAL D5	0.57	**
	nose D7	-0.15	ns
	BAL D7	-0.01	ns
**Neutrophils in BAL**	(%) D5	-0.11	ns
	(10^6/L) D5	0.24	ns
	(%) D7	0.49	ns (0.07)
	(10^6/L) D7	-0.28	ns

R, Pearson’s correlation coefficient

BAL, bronchoalveolar lavage; nose, nasal secretions collected on day (D)5 or D7 post-infection

Significant differences between treatment groups are indicated by asterisks (p<0.05 (*) and p<0.01 (**)). P-values within 0.05 ≥ p ≤ 0.1 are given within brackets.

### Aspirin and meloxicam induced a shift in eicosapentaenoic acid-derived oxylipids in BAL

**Fig 9:**
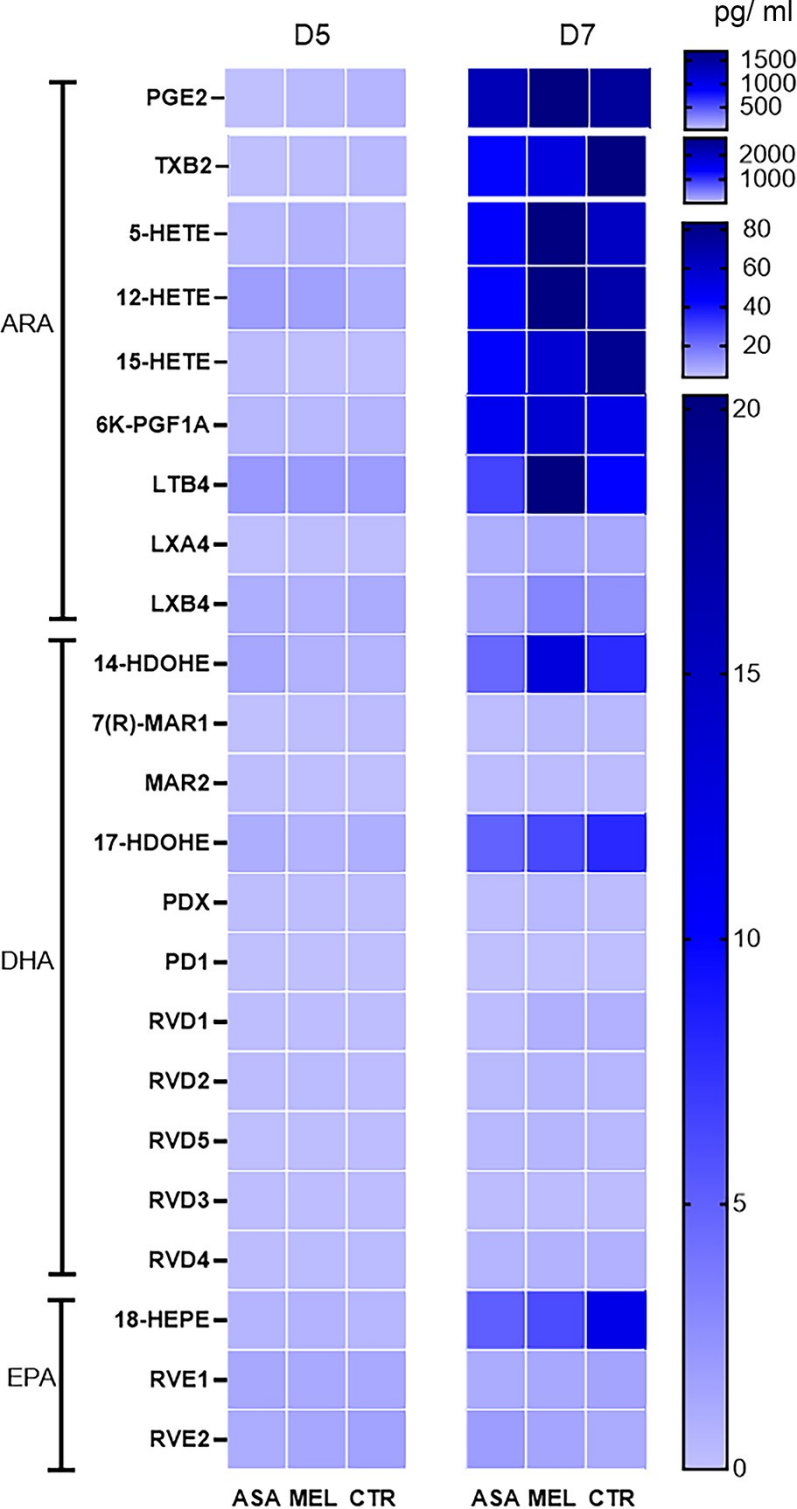
Oxylipids in bronchoalveolar lavage of BRSV-infected, aspirin- or meloxicam-treated calves and controls. Mean concentration of oxylipids detected by liquid chromatography tandem mass spectrometry with triple quadrupole mass detectors in bronchoalveolar lavage (BAL) collected on day (D)5 and D7 post infection. Please note that the scales differ.

Results of oxylipid analyses of BAL that were run by the two laboratories were in excellent agreement for leukotriene B4 (LTB4), PGE2, thromboxane B2 (TXB2) and 15-hydroxyeicosatetraenoic acid (HETE, Spearman correlation coefficient 0.85–0.98), but less for 12-HETE (Spearman correlation coefficient 0.63). The infection induced significantly higher concentrations of all oxylipids on D7 compared to on D5 (0.000 ≥ p ≤0.026, Fig 9), except for RVE1 and RVE2, for which the increase was not significant (S1 Fig).

On D7, arachidonic acid-derived precursor metabolites 12- and 15-HETE as well as the docosahexaenoic acid-derived precursor metabolites 14- and 17-hydroxydocosahexaenoic acid (HDOHE) and the eicosapentaenoic acid-derived precursor metabolite 18-hydroxyicosapentaenoic acid (HEPE) were detected at higher average concentrations than their end products (SPM: LXA4 and LXB4, maresins, MAR, protectins, PD and resolvins, RV, Fig 9). By using the mixed model effect for repeated measures, corrected for multiple comparisons, treatment significantly affected the concentration of RVD1 (p = 0.006) and tended to do so for RVD2 (p = 0.091). The following differences were significant between treatment groups on the same day: meloxicam-treated calves had significantly higher concentrations of RVD1 than aspirin-treated calves on D7 (and tented to have so for RVD2) and aspirin-treated calves had first significantly lower, and then significantly higher concentrations of RVE2 than controls on D5 and D7, respectively (S1 Fig, due to the different effect of aspirin on different days compared to no treatment, the overall effect of treatment on RVE1 was not significant).

The concentrations of 15-epi-LXA4, 17-epi-RVD1, 17-epi-RVD3 and 17-epi-PD1, which belong to aspirin-triggered specialised pro-resolving mediators, were very close to, or below, the limit of detection and did not differ significantly between calves of the different groups (S1 Fig).

### The oxylipid pattern in plasma differed from that in BAL

**Fig 10:**
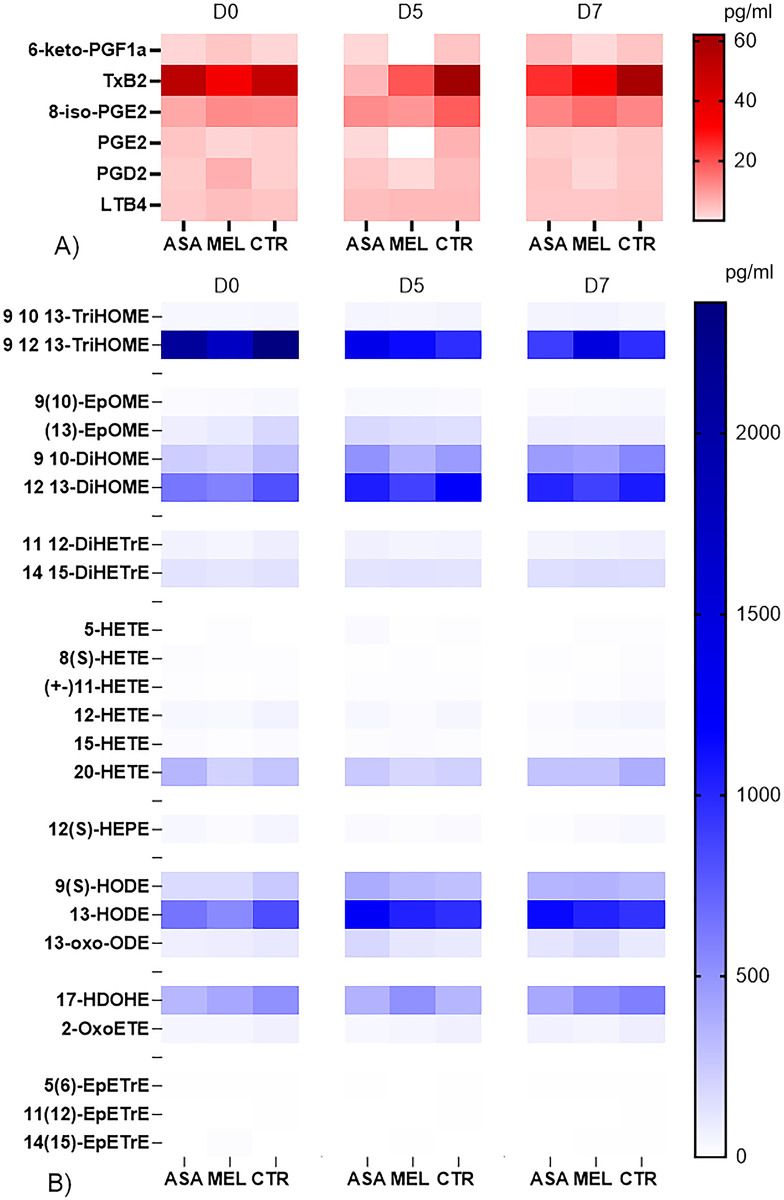
Oxylipids in plasma of BRSV-infected, aspirin- or meloxicam-treated calves and controls. Mean concentrations of A) prostaglandins, leukotriene and thromboxane, and B) other oxylipids detected by liquid chromatography tandem mass spectrometry with triple quadrupole mass detectors in plasma collected on day (D)0, D5 and D7 post infection.

In general, oxylipids were detected at lower levels in plasma than in BALs (Figs 9 and 10). For example, the highest detected concentration of PGE2 was 14 pg/ml in plasma and 467 pg/ml in BAL (D5 and D7, respectively, in the control calf 14) and the highest detected concentration of TXB2 was 144 pg/ml in plasma and 4567 pg/ml in BAL (D5 and D7, respectively, in the control calf 12). The overall kinetics of TXB2, PGs and leukotriene B4 in plasma is presented in S2 and S3 Figs. Despite that 29 oxylipids were quantified in plasma, only a few differed significantly between treated calves and controls.

The following significant differences were detected by using the mixed model effect for repeated measures and by correcting for multiple comparisons: meloxicam-treated calves had significantly lower plasma concentrations of 6-keto-PGF1α (a stable metabolite of PGI) than controls on D5 (p = 0.001) and aspirin-treated calves had significantly lower plasma concentrations of TBX2 and 12(S)-HEPE than controls on D5 (p = 0.019) and D7 (p = 0.025), respectively.

## Discussion

This study investigated the effects of NSAIDs administered early in the course of a BRSV infection in calves. The experiment was designed to mimic a situation in which medication is administered to prevent inflammation before it establishes. With this approach, neither aspirin, nor meloxicam reduced clinical signs or lung lesions induced by BRSV. In contrast, relative to untreated controls, meloxicam appeared to induce a downregulation of proteins related to immunoglobulins, and an augmented neutrophil influx in BAL in response to BRSV. Due to the small number of animals included in the study, this would need to be further explored in a larger study population including different genetics.

Although a rapid neutrophil response is likely to lead to virus neutralisation and protection against clinical signs, a neutrophil activity that is too strong and prolonged is associated with exaggerated RSV disease (through obstruction of airways and destruction of lung elastin fibres by neutrophilic products [34]). The increased neutrophil influx in the lungs of the meloxicam-treated calves agrees with previous studies. In one study, flunixin meglumine induced extensive pulmonary neutrophil infiltration in *Mycoplasma bovis*-infected, enrofloxacin-treated calves [35]. Moreover, in cows, meloxicam induced earlier and stronger average milk somatic cell count compared to placebo after LPS challenge [36]. Both LPS and viruses such as RSV and SARS-CoV2 act as toll-like receptor 4 (TLR4) agonists, which stimulate neutrophil migration and formation of NETs, and may therefore induce an exaggerated inflammation [19, 34, 37–39]. This reaction is further augmented in over-conditioned individuals, through macrophage infiltration of tissues and cytokine production [40, 41].

The relative abundance of neutrophil-related proteins in BAL, such as resistin and myeloperoxidase, was linearly associated with the extent of lung lesions. Although the small animal number affected the reliability of this analysis, this finding is in agreement with previous studies using a similar model [20]. Resistin sensitises neutrophils to TLR4 activation by LPS, to induce NET formation and pro-inflammatory cytokines [42], and since the fusion protein of RSV similarly activates TLR4 [19], resistin is probably important in RSV pathogenesis. The plasma concentrations of resistin increases during lipolysis in the early lactation of dairy cows [43] and this may contribute to the sensitivity of these individuals to both bacterial and primary BRSV infections.

The Liver X Receptor/ Retinoid X Receptor (LXR/RXR) pathway, the Farnesoid X Receptor (FXR)/RXR pathway and the 24-Dehydrocholesterol Reductase (DHC24) signaling pathway were highly significantly downregulated in meloxicam-treated calves relative to controls in BAL on D7. These are all involved in cholesterol metabolism and are activated in macrophages and hepatocytes through TLR4 stimulation, such as by BRSV, or during negative energy balance [44–46].

A potential adverse effect of meloxicam is the inhibition of negative feedback systems in inflammation. The downregulation of the LXR/RXR pathway in meloxicam-treated calves may partly explain the reinforcement of the neutrophilic responses to BRSV. Indeed, in mice, LXR controls Th17 responses and neutrophil recruitment to the lung and induces an anti-inflammatory macrophage phenotype, with diminished production of cytokines involved in exaggerated inflammation [47, 48]. In addition, LXR augments insulin-sensitisation and the intracellular concentrations of lipids with anti-inflammatory effects, such as 20:5n3 (eicosapentaenoic acid, EPA) and 22:6n3 (docosahexaenoic acid, DHA) produced from 18:3n3 (alpha-linolenic acid, ALA) [40]. The intracellular lipid and lipoxid concentrations were unfortunately not measured in the present experiment, due to limitations in the funding.

Neutrophils are not only activated through TLR-4 stimulation, but also by a multitude of additional systems, such as by isoprostanes produced during oxidative stress [49], complement factors (C3a, C5a), cytokines (*e*.*g*. IL-8, IL-17), bradykinin [50] and leukotriene B4 [51]. Single proteins involved in the complement and the bradykinin response (C4, KNG1) were downregulated in meloxicam-treated animals, and leukotriene B4 did not increase significantly compared to controls, in contrast to what was previously reported for meloxicam in cattle [52] and for aspirin, ibuprofen and meloxicam in humans [53–55]. Leukotriene B4 acts as a chemoattractant for neutrophils and primes neutrophils to an activated state, but it is rapidly metabolised, and thus difficult to quantify over time [56, 57]. In future studies, it might therefore be more useful to study cysteinyl leukotriene in the urine, as performed in studies of human asthma [58].

The inflammatory phase, during which neutrophils perform phagocytosis and release NETs, must rapidly be succeeded by a resolution phase, during which further neutrophil infiltration is stopped and dead cells are removed (reviewed by [59]). The resolution is driven by PGE2, PGI2 and 15-PGJ, which activate EP4 receptors on macrophages in the airways [60] and induce SPM production, neutrophil apoptosis and efferocytosis [15]. In addition, PGE2 and PGI2 regulate the pulmonary endothelial barrier integrity, decrease the vascular and bronchial resistance and induce re-epithelialisation [16–18, 61]. Overall, PGE2 was detected in high concentrations relative to other oxylipids in BAL, but the concentrations did not differ between treated calves and untreated controls. However, meloxicam reduced the plasma concentrations of 6-keto-PGF1α, a stable metabolite of PGI2. This may have contributed to an activation of neutrophils, but did not seem to suppress the induction of RVD1 and RVD2 in BAL.

Because the study terminated on D7, the resolution of inflammation could not be studied in detail. Consequently, the precursors were consistently detected in higher amounts than the corresponding SPMs. Although concentrations of different SPMs were very low and untreated calves had only limited lung lesions and therefore no reason for healing, RVD1 was induced at higher concentrations in meloxicam-treated calves than in aspirin-treated calves. At this dose-regimen and timing of sampling, aspirin-triggered SPMs could not be detected. In humans, a low, but not high doses of aspirin induced aspirin-triggered 15-epilipoxin A4 [61 mg, but not 325 mg or 650 mg aspirin *in toto*, *i*.*e*. 1, but not 5 or 9 mg/kg at 70 kg BW] [62]. It is therefore possible that the aspirin dose used herein, which was based on a previous study in cattle [63], was too high to induce aspirin-triggered SPMs.

Both aspirin and meloxicam affected oxylipid concentrations in plasma, possibly because the drugs were administered parenterally. The intravenous route of drug administration was chosen because it generates less individual variation in plasma concentration than the subcutaneous or oral route in calves [64, 65] and aspirin was administered twice because, although it acetylates the COX enzymes in an irreversible manner, it is rapidly cleared from the circulation [63]. In contrast, as therapeutic plasma concentrations of meloxicam can remain in calves for several days [64], a single dose was applied.

The downregulation immunoglobulin-related proteins in meloxicam-treated calves agrees with one study, in which meloxicam reduced the IgM, IgG and serum neutralising responses six days post SARS-CoV2-infection in mice [66], but disagrees with another, in which meloxicam induced improved antibody responses against vaccination immediately after international transport of beef cattle [67]. In the present study, the early detection of immunoglobulin-related proteins in controls indicates that these were parts of natural antibodies, which are mostly of the IgM isotype and which recognise conserved structures in a polyreactive manner. Natural antibodies are potent activators of the complement system and, when aggregated to complement factors and antigen, initiate adaptive humoral responses efficiently (reviewed by [68]).

Calf 10, the calf with the highest sum of clinical scores, the highest quantity of BRSV-RNA in BAL on D5, the highest lung neutrophil score and the most extensive lung lesions on D7, was the only calf in which immunoglobulin lambda was not detected on D5. A meloxicam-induced immunoglobulin lambda downregulation in this calf may have contributed to an insufficient protection of the lower airways and a strong neutrophil response induced by BRSV, which probably contributed to clinical signs and lesions, but also to the limitation of further virus replication in the lung.

In summary, treatment with meloxicam or aspirin early in the course of a BRSV-infection did not decrease clinical signs or the extent of lung lesions induced by BRSV. Meloxicam induced an increased proportion of pulmonary neutrophils and downregulation of proteins that were likely included in natural antibodies in BAL. The extent of lung lesions was associated to the early replication of BRSV, and to the relative abundance of neutrophil-related proteins in BAL. These results do not support the use of NSAID early in the course of a BRSV-infection in cattle.

## Supporting information

S1 Table Bacteria detected in bronchoalveolar lavage D7 post mortem. (DOCX)

S2 Table List of differentially expressed proteins in BAL. (XLSX)

S1 Fig Specialised proresolving mediators in BAL. (TIF)

S2 Fig Kinetics of TBX2 in plasma. (TIF)

S3 Fig Kinetics of prostaglandins in plasma. (TIF)

S4 Fig Kinetics of oxylipids in plasma. (TIF)
