# Supplementary material for: Effects of early treatment with nonsteroidal anti-inflammatory drugs (NSAIDs) on the bronchoalveolar lavage proteome and oxylipids during bovine respiratory syncytial virus (BRSV) infection
Source: PLoS One. 2024 Nov 15;19(11):e0309609. doi: 10.1371/journal.pone.0309609 (PMC11567528; doi:10.1371/journal.pone.0309609)
Supplement: S1 Table — (DOCX) [file pone.0309609.s001.docx]

**Supplement (S)1 Table. Bacteria detected in bronchoalveolar lavage of BRSV-infected meloxicam-or aspirin-treated calves and untreated controls (no. 1-15), D7 *post mortem***

| ASA | MEL | CTR |
| --- | --- | --- |
| 1. ND | 6. Sparse Str spp. | 11. ND |
| 2. ND* | 7. Sparse Str spp. | 12. Sparse Paenibacillus spp. |
| 3. ND* | 8. Sparse Str spp. | 13. ND |
| 4. Sparse Str spp. | 9. Sparse Staph spp. | 14. Sparse Str spp.* |
| 5. ND* | 10. ND* | 15. Sparse Paenibacillus amylolyticus |

ND; no bacterium detected, *; calf in which the D-1 BAL was unsuccessful
