# Supplementary figures and images for: Effects of early treatment with nonsteroidal anti-inflammatory drugs (NSAIDs) on the bronchoalveolar lavage proteome and oxylipids during bovine respiratory syncytial virus (BRSV) infection
Source: PLoS One. 2024 Nov 15;19(11):e0309609. doi: 10.1371/journal.pone.0309609 (PMC11567528; doi:10.1371/journal.pone.0309609)

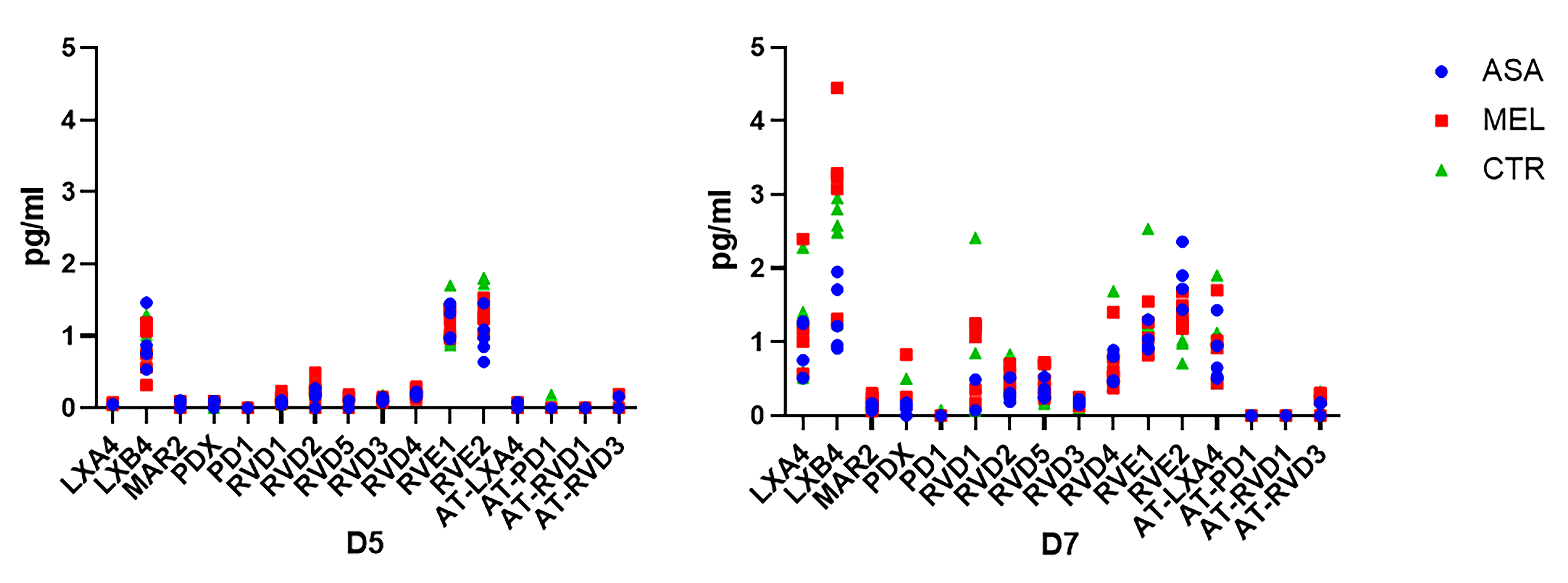

Supplement: S1 Fig — (TIF) [file pone.0309609.s003.tif]
